# Patterns and driving factors of ecological stoichiometry in system of deadwood and soil in mountains forest ecosystem

**DOI:** 10.1038/s41598-023-32946-1

**Published:** 2023-04-07

**Authors:** Ewa Błońska, Wojciech Piaszczyk, Jarosław Lasota

**Affiliations:** Department of Ecology and Silviculture, Faculty of Forestry, University of Agriculture in Krakow, 29 Listopada 46 Str., 31-425 Kraków, Poland

**Keywords:** Biogeochemistry, Ecology, Biogeochemistry, Forest ecology

## Abstract

The aim of our research was to identify the factors that most strongly determine the C, N and P cycles in the deadwood—soil system in mountains forest ecosystems. We assumed that the climatic conditions resulting from the location in the altitude gradient and rate of deadwood decomposition most strongly determine the C/N/P stoichiometry. A climosequence approach comprising north (N) and south (S) exposure along the altitudinal gradient (600, 800, 1000 and 1200 m a.s.l.) was set up. Spruce logs at different decomposition stages (III, IV and V) were selected for the analysis in Babiogórski National Park (southern Poland). We calculated the C/N/P stoichiometry for deadwood and soil samples to reflect the nutrient availability. Our research indicates a very strong influence of the location conditions in the altitude gradient on the C/N/P stoichiometry. The GLM analysis confirmed the importance of high elevation in shaping the C, N and P content. A strong correlation was confirmed between P content, N content and C/N ratio. A higher C/N/P ratio was found in deadwood compared to soil, regardless of location. Decaying wood is an important source of N and P and the degree of decomposition made a significant contribution to explaining the variability of C, N and P content. The obtained results indicate the need to leave deadwood in forest ecosystems in order to improve biogeochemical cycles. Deadwood, by having a beneficial effect on many components of the forest ecosystem, will improve its biodiversity and, consequently, its stability.

## Introduction

Deadwood occurs in every forest ecosystem and performs many functions on which their proper functioning depends^1,2^. The key functions of deadwood include increasing biodiversity, shaping microhabitats and soil properties, storing nutrients and water^3–5^. Deadwood plays an important role in the cycle of carbon, nutrients and hydrological cycles^6–8^. Decaying deadwood is an important component of biodiversity in European forests and is used as an indicator in assessing and monitoring the biodiversity of forest ecosystems.

Deadwood can vary in type and degree of decay. The most deadwood can be divided into coarse woody debris (CWD) and fine woody debris (FWD)^9^. Deadwood includes dead woody plant material, for example standing dead trees, lying dead trees, snags, stumps and branches^10^. In studies of deadwood it is important to assessing the degree of deadwood decomposition and this is determined using a five-stage classification based on the characteristics of the wood, such as the presence of bark, structure and color of the wood^11^. Depending on the degree of decomposition, wood affects the soil environment^12^. More heavily decomposed wood has a greater impact on physical, chemical and biochemical properties compared to less decomposed wood^13^. The rate of decomposition of deadwood depends on the type of wood, species and conditions in which the decomposition process takes place^14^. Thermal conditions and moisture have a very strong impact on the rate of decomposition in forest ecosystems^15–17^. According to Liu et al.^18^ warming affects the circulation of C, N and P in the forest ecosystem. The decomposition process is important for nutrient cycling in terrestrial ecosystems and is influenced not only by climate but also by microorganisms^19,20^. Mountain forest ecosystems growing in different climate conditions provide the opportunity to conduct research on the rate of decomposition depending on climatic conditions^20,21^. In addition to the temperature along the elevation gradient. The water regime changes, which has a direct impact on the rate of decomposition^22^.

The rate of decomposition of organic remains in forest ecosystems determines the cycle of C, N and P. Ecological stoichiometry is used to represent the balance between many elements, with particular emphasis on C, N and P^23^. C/N/P stoichiometry is closely related to elementary processes such as photosynthesis, respiration or mineralization of organic matter^24^. According to Liu et al.^25^ the C/N/P stoichiometry strongly correlates with thermal conditions and the amount of precipitation. In the research of Chen et al.^26^ the climatic factor made a strong contribution to explaining the C/N/P stoichiometry of soils. Deadwood is an important element of forest ecosystems, it affects the C, N and P cycles and thus recognition the patterns of ecological stoichiometry in the deadwood—soil system will allow for a better understanding of nutrient cycling, ecosystem dynamics and mechanisms of the biogeochemical cycle. So far, there have been no research on C/N/P stoichiometry in the deadwood-soil system which would consider the influence of climatic conditions.

In our study, we used the climosequence approach in determining patterns of ecological stoichiometry in system of deadwood and soil. The aim of our research was to identify the factors that most strongly determine the C, N and P cycles in the deadwood—soil system. We assumed that the climatic conditions resulting from the location in the altitude gradient most strongly determine the C/N/P stoichiometry. We assume that the greatly decomposed wood in the 5th stage of decomposition strongly affects the circulation of C, N and P.

## Materials and methods

### Study area

The research was carried out in the Babiogórski National Park in southern Poland. (49°35′18′′N; 19°32′23′′E). The study included three transects with study plots on the southern and northern slopes with an inclination of 15°. A climosequence approach comprising north (N) and south (S) exposure along the altitudinal gradient (600, 800, 1000 and 1200 m a.s.l.) was set up. In each variant of the elevation, in each transect research plots were designated separately for deadwood in different decay rate. Lying dead trees of spruce in the form of logs at different decomposition stages (III, IV and V) were selected for the analysis. Our study covered logs in the III-V degree of decomposition with visible signs of the decomposition process. Logs in the first and second stages of decomposition are not included in this study as previous studies have confirmed that they have no or only a small impact on soil characteristics^27,28^. The decay classes (DC) of logs were estimated according to the classification of dead trees in Maser et al.^29^ which was used in the previous study^4,5^. The tested soils were characterized by the similar texture (average sand content was 52%, silt 42% and clay 6%). The sensors (5TM, Sensor, Decagon) were used to temperature test during the year. The average annual temperature of the S exposure for the study plots at 600 m a.s.l. it was 6.8 °C, for the study plots of 800 m a.s.l. it was 5.6 °C, at an elevation of 1000 m a.s.l. it was 4.5 °C and at an elevation of 1200 m a.s.l. it amounted to 3.4 °C. The average annual temperature of the N exposure for the study plots at 600 m a.s.l. was 6.0 °C, for the study plots of 800 m a.s.l. was 5.1 °C, at an elevation of 1000 m a.s.l. was 4.0 °C and at an elevation of 1200 m a.s.l. was 3.1 °C. Regardless of the exposure, the lowest locations (600 m a.s.l.) are characterized by lower average annual precipitation (about 1200 mm), while at an altitude of 1200 m a.s.l. the average annual precipitation is about 1400 mm^30^. The study plot was the area with the log covered by the analysis with the soil sampling area around it. We selected logs with diameter between 25 and 35 cm to ensure direct comparability of observations. The study covered 72 logs of deadwood located in different location condition (2 exposure × 4 points in altitude gradient × 3 degrees of decomposition × 3 repetitions = 72 logs). Wood samples measuring 7 × 7 × 7 cm for laboratory analysis were taken from the midpoint of each log. In our study we analyzed 144 soil samples. The soil samples were collected directly under the log, the soil was sampled from 0 to 10 cm depth using a small spade. Additionally soil samples (background) were taken 1 m from logs from 0 to 10 cm depth. In total, 216 wood and soil samples were taken for laboratory tests. In the study, we included samples from decaying logs (D), soil samples lying under deadwood (UD) and soil samples taken 1 m from the logs of decaying wood (C). The field study was realized in 2021.

### Laboratory analysis

Total nitrogen and carbon content was determined using a LECO CNS True Mac Analyser (Leco, St. Joseph, MI, USA). We used the ICP analysis (ICP-OES Thermo iCAP 6500 DUO, Thermo Fisher Scientific, Cambridge, U.K.) to determine the P content. We determined the levels of P after mineralisation of the mixture with concentrated nitric and perchloric acids at a ratio of 3:1 and calculated the C/N, C/P and N/P ratios on a molecular level.

### Statistical analysis

The Spearman correlation coefficient between the C, N and P content and C/N/P stoichiometry of deadwood and soil was calculated. The Shapiro–Wilk test was used to assess normality and Levene’s test was used to check the homogeneity of variances. Regression models were developed for the N, P content and C/N ratio with a division into three types of samples (wood, soil under wood and control). In order to show the relationship between the examined variables, principal component analysis (PCA) was performed. The GLM analysis was used to determine the significance of the influence of altitude, exposure, samples type and the degree of decomposition of deadwood on the C/N/P stoichiometry. Differences with *p* < 0.05 were considered to be statistically significant. All statistical analyses were performed using R statistical software (R Core Team 2020), R Studio (RStudio Team 2020), and Statistica 10 software (2010).

## Results

The GLM analysis confirmed the influence of altitude, decomposition degree and type of sample on the content of N, C and P (Table 1). In the case of the P content, the exposure also had a significant impact on its content. For the N and C content, the exposure had no significant effect (Table 1).

**Table 1:** GLM analysis for C, N, P content and C/N/P stoichiometry.

	C		N		P		C/N		C/P		N/P	
	F	p	F	p	F	p	F	p	F	p	F	p
Exposure (E )	0.01	0.9418	0.01	0.9378	12.46	**0.0005**	1.62	0.2048	4.12	**0.0444**	7.61	**0.0066**
Altitude (A)	8.47	**0.0001**	8.40	**0.0001**	7.59	**0.0001**	2.16	0.0950	4,23	**0.0068**	4.65	**0.0041**
Decay clas (DC)	4.42	**0.0001**	7.61	**0.0001**	4.18	**0.0001**	5.74	**0.0001**	1.94	0.0922	4.21	**0.0032**
Samples type (ST)	55.35	**0.0000**	36.28	**0.0000**	66.36	**0.0000**	36.80	**0.0000**	25.83	**0.0000**	46.97	**0.0000**
E × A	11.57	**0.0001**	11.84	**0.0001**	10.10	**0.0000**	5.18	**0.0021**	3.67	**0.0140**	4.26	**0.0066**
E × DC	10.50	**0.0001**	2.55	0.0814	11.16	**0.0001**	1.02	0.3619	2.52	0.0839	7.21	**0.0011**
E × ST	2.16	0.1185	4.57	**0.0120**	7.33	**0.0009**	5.89	**0.0035**	9.35	**0.0001**	2.50	0.0856
A × DC	3.78	**0.0017**	1.62	0.1447	3.67	**0.0020**	1.88	0.0885	2.37	**0.0328**	0.88	0.5071
A × ST	11.62	**0.0000**	6.03	**0.0001**	3.86	**0.0014**	2.23	**0.0441**	4.31	**0.0005**	8.86	**0.0000**
DC × ST	4.59	**0.0118**	5.00	**0.0081**	4.19	**0.0172**	29.41	**0.0000**	14.52	**0.0000**	4.28	**0.0158**
E × A × DC × ST	1.43	0.1591	1.74	0.0664	4.22	**0.0000**	2.16	**0.0171**	3.61	**0.0001**	2.84	**0.0017**

Significance effect (*p* < 0.05) are show in bold.

**Figure 1:**
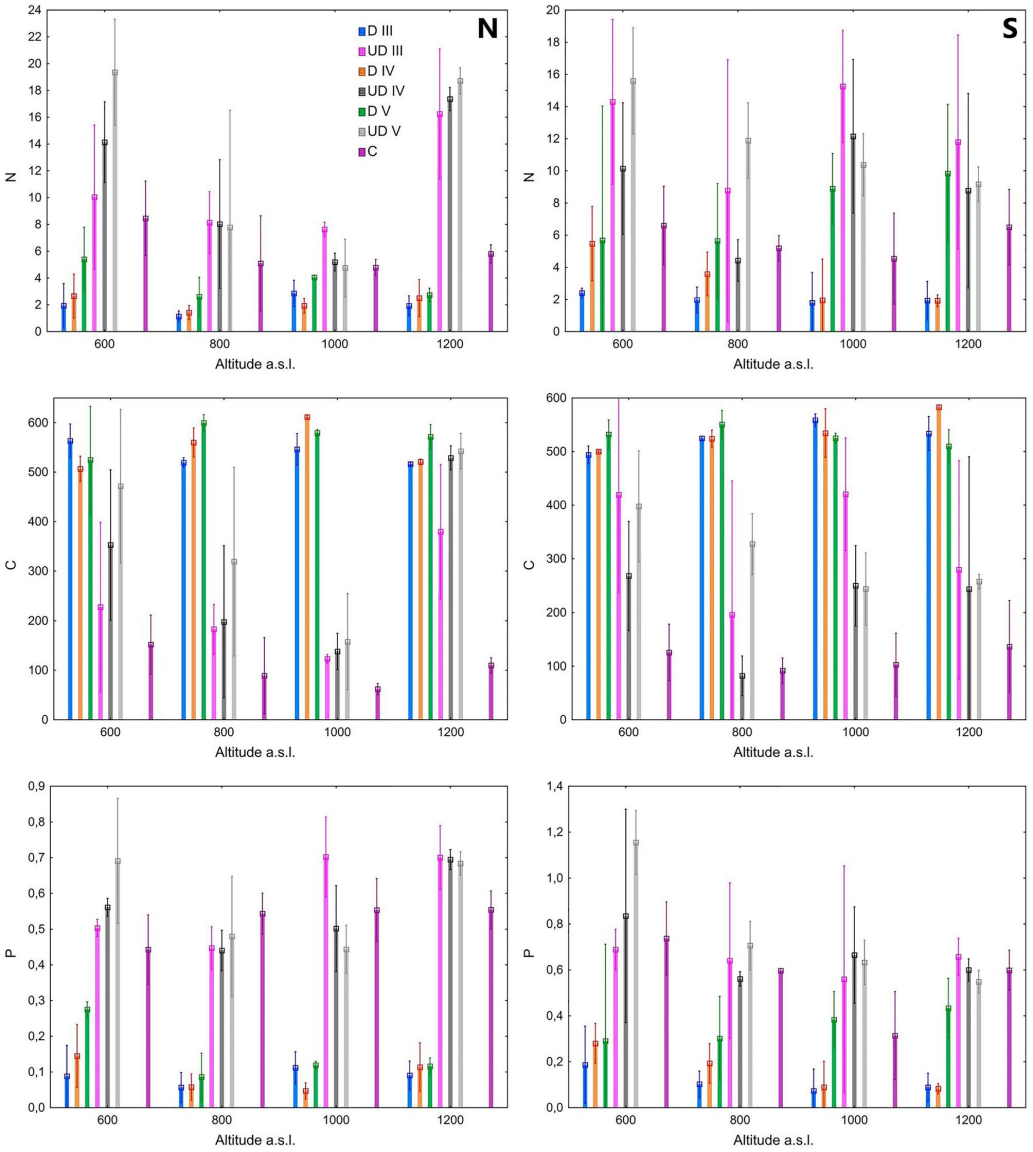
Content of N (g kg^-1^), C (g kg^-1^) and P (g kg^-1^) in wood (D) and soil samples (*UD* soil samples under deadwood; *C* control soil) in relation to exposure, altitude and decay clases (III, IV and V).

The highest nitrogen content was recorded in soils affected by the most strongly decomposed wood in the 5th stage of decomposition at an altitude of 600 m a.s.l. at the N and S exposure (19.35 g kg^-1^ and 15.59 g kg^-1^ respectively). Regardless of the location and the climate conditions prevailing there, the soil affected by the impact of decaying wood is characterized by a higher content of N and C (Fig. 1). Wood in the 5th degree of decomposition is characterized by the highest content of N regardless of the location (Fig. 1). At the N and S exposure, the soil affected by the wood had a higher C content compared to the control soil. The content of C in soils under the influence of wood in the 5th degree of decomposition at the N exposure ranged from 157.6 to 542.5 g kg^-1^, and in the samples of control soils the carbon content ranged from 61.9 to 152.1 g kg^-1^. The content of C in soils affected by wood in the 5th degree of decomposition at the S exposure ranged from 243.5 to 397.9 g kg^-1^, and in the samples of control soils, the content of C ranged from 91.7 to 136.5 g kg^-1^. In most locations, the impact of decaying wood on shaping the phosphorus content in soils influenced by wood can be seen. In most cases, the content of phosphorus in soils affected by decaying wood was higher than in control soils. At the N exposure at the altitude of 600 m a.s.l. lower P content was noted in the soils affected by the decaying wood compared to the S exposure (Fig. 1).

**Figure 2:**
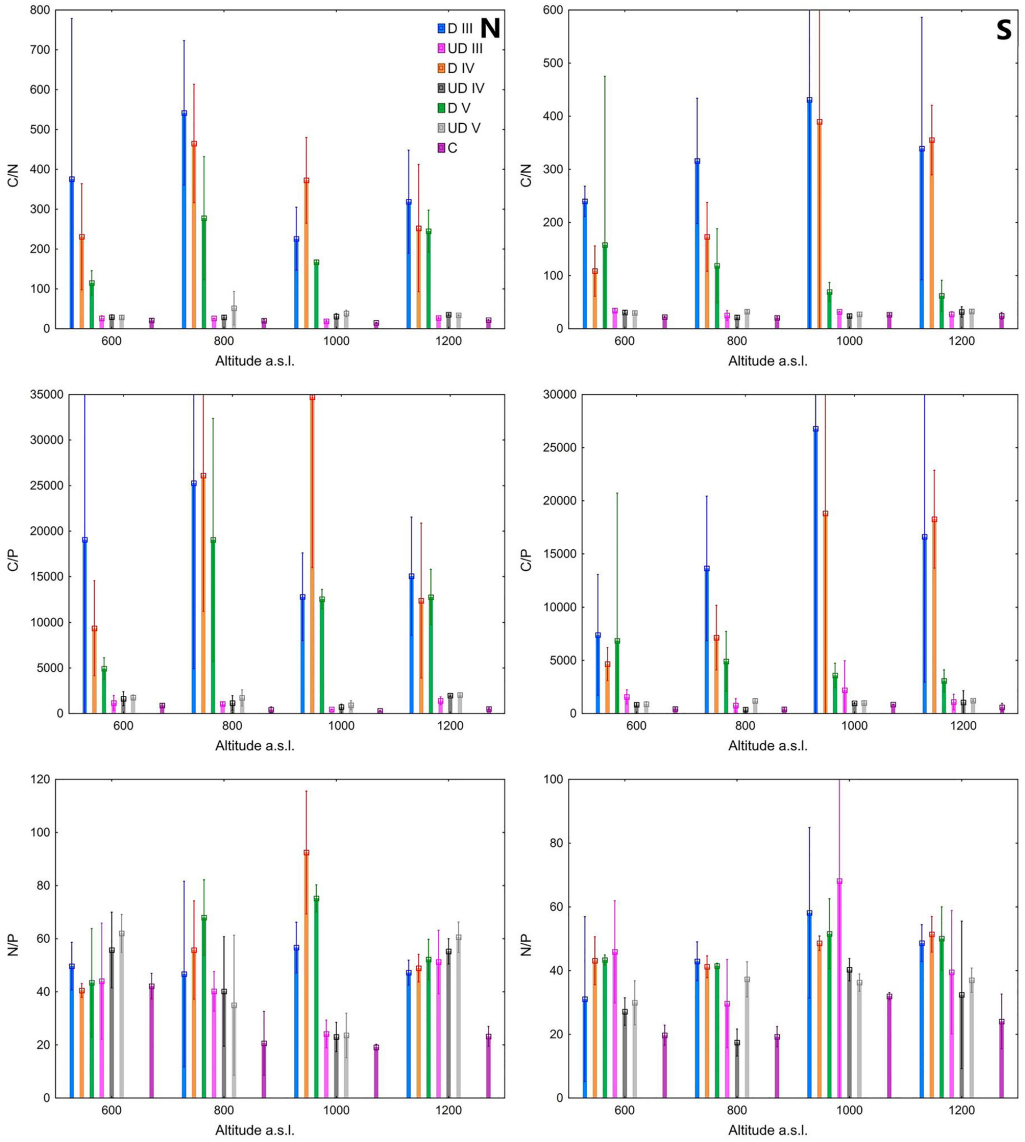
C/N/P stoichiometry of wood (D) and soil samples (*UD* soil samples under deadwood; *C* control soil) in relation to exposure, altitude and decay clases (III, IV and V).

The conducted GLM analysis confirmed the importance of the degree of decomposition in shaping the C/N ratio. In the case of C/P, the location conditions were more important than the degree of decomposition (Table 1). Wood samples, regardless of the degree of decomposition, were characterized by higher C/N and C/P ratios compared to the soil samples (Fig. 2). The average C/N of less decayed wood (III DC) was in the range of 225.9–541.7, the C/N of wood in IV DC was from 108.6 to 464.8, and the C/N of the most heavily decayed wood in the V degree of decay was in the range of 61.8–277.4. As in the case of the C/N ratio, the C/P ratio decreased in more strongly decomposed wood, regardless of the location. Soils affected by decaying wood had higher C/N and C/P ratios compared to the control. In the case of the N/P ratio, the GLM analysis confirmed the importance of the location conditions and the degree of decomposition in shaping its value. Regardless of the location, the N/P ratio was higher in the wood samples and in the soil affected by decaying wood compared to controls (Fig. 2).

**Figure 3:**
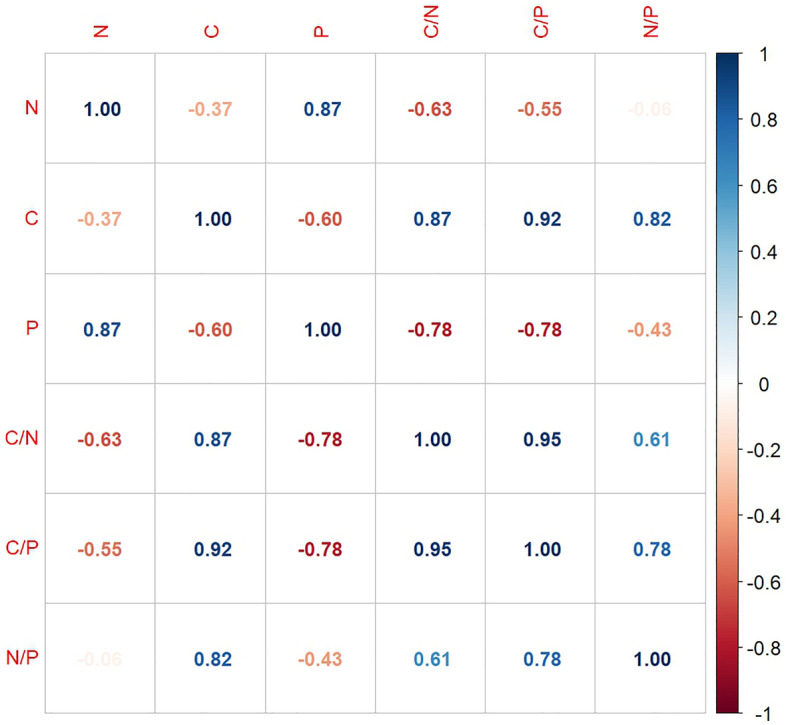
Correlation between content of N, C and P and stoichiometry C/N/P in wood and soil samples.

The conducted correlation analysis indicates a strong positive relationship between the content of N and P (r = 0.87) (Fig. 3). P content negatively correlated with C content, C/N ratio and C/P ratio (r =  − 0.60, r =  − 0.78, r =  − 0.78 respectively). The C/N ratio strongly positively correlated with the C/P and N/P ratios (r = 0.95 and r = 0.61, respectively). In addition, there was a strong positive relationship between the C/P and N/P ratios (r = 0.78) (Fig. 3).

**Figure 4:**
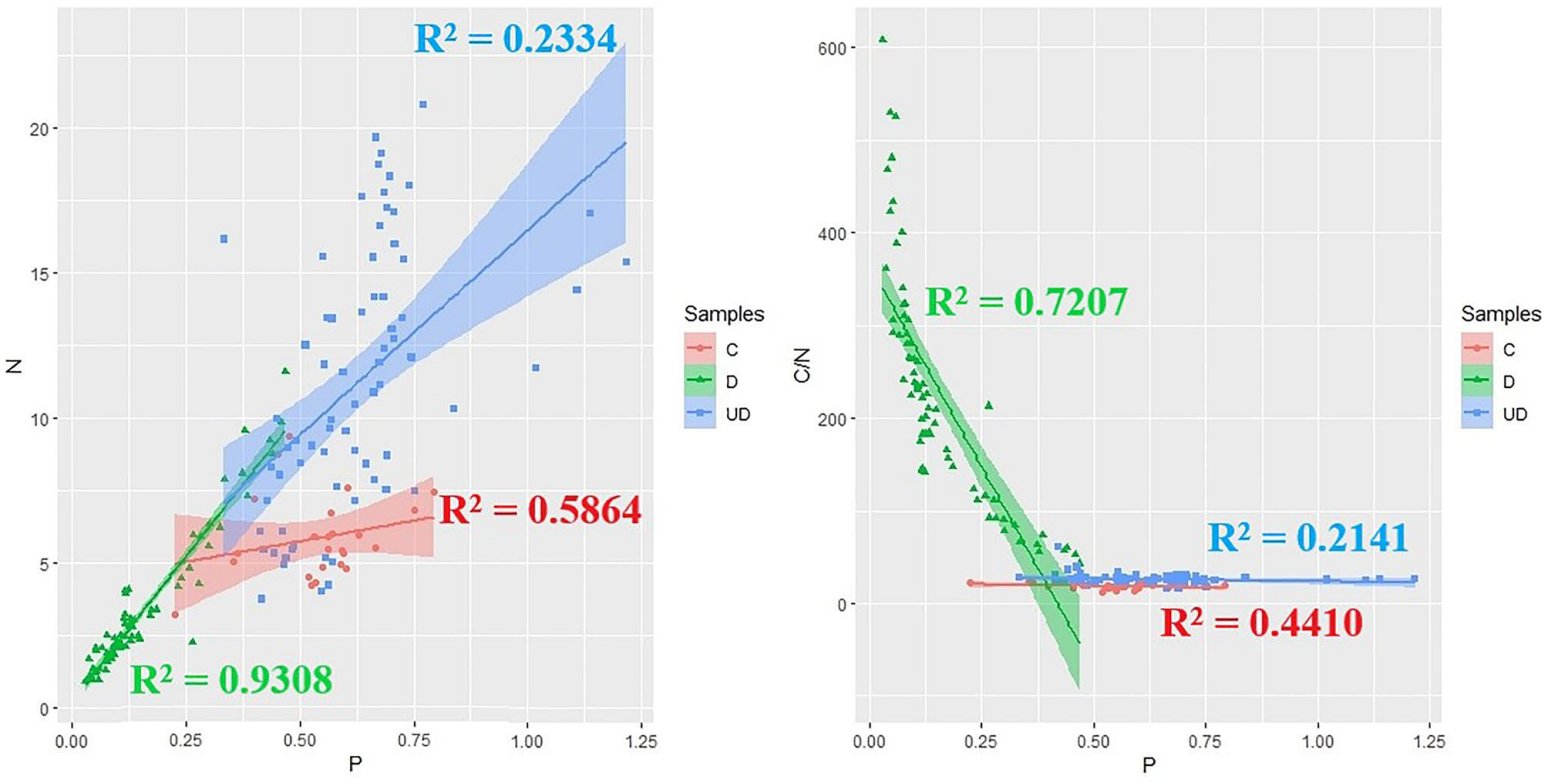
Regression lines between N and P content and between C/N ratio and P content in relation to samples types.

Figure 4 shows the relationship between the content of N and P in relation to the type of sample. In the case of both wood and soil samples, the P content increases with increasing N content. In the case of wood samples, a strong negative relationship was noted between the C/N ratio and the P content (Fig. 4).

**Figure 5:**
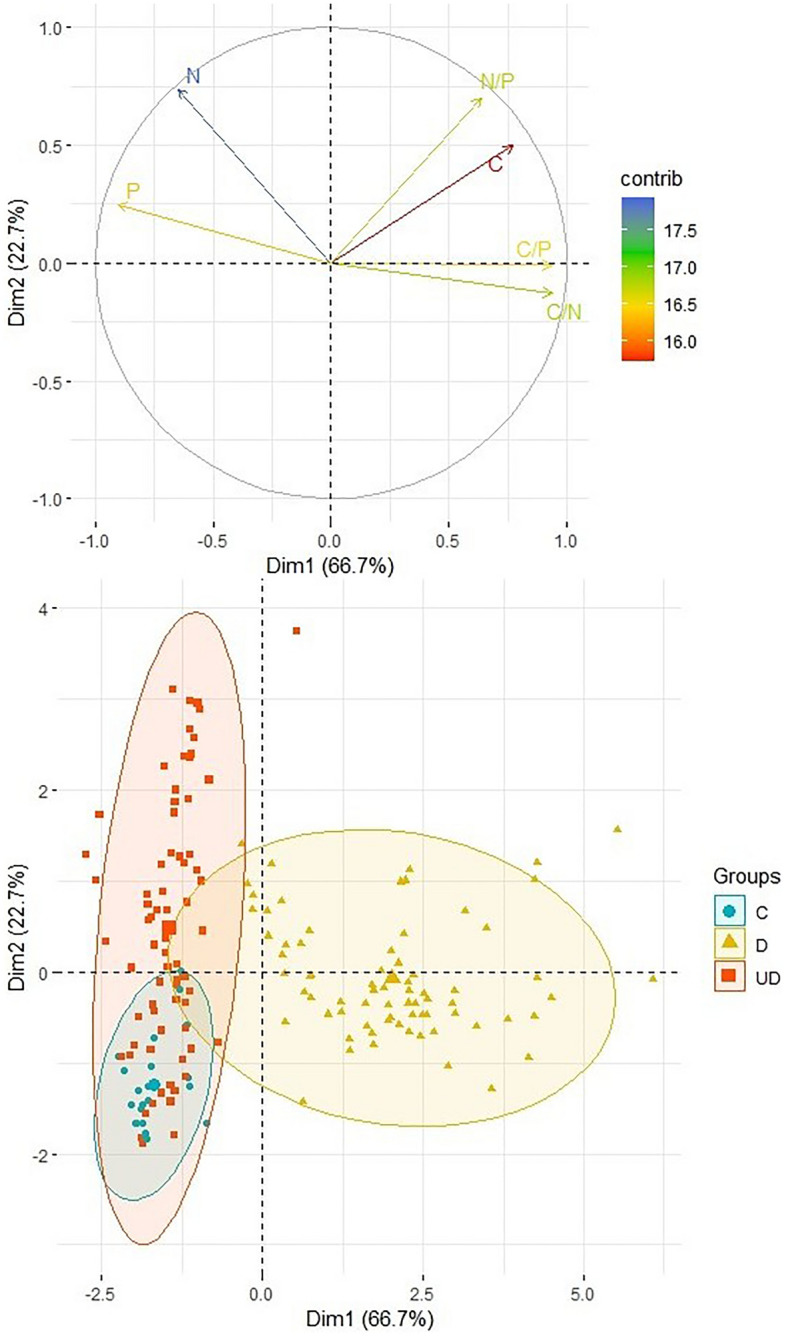
Projection of variables on the plane of the first and second PCA factors (*C* control samples, *D* deadwood, *UD* soil under deadwood).

The performed PCA analysis confirmed the relationship between the studied properties (Fig. 5). Two main factors contributed to the observed variance (89.4%): factor 1 accounts for 66.7% of the variance while factor 2 explains 22.7% of the variance. Factor 1 is related to P content and C/P and C/N stoichiometry, while factor 2 is related to N content and N/P ratio. The performed PCA analysis confirms the positive relationship between the P and N content and the positive relationship between the C content and the C/N, C/P and N/P stoichiometry. The PCA analysis confirmed that the wood samples are characterized by a higher C/N/P stoichiometry compared to the soil samples. In terms of the examined properties, the wood samples form a separate set. The highest content of N and P was recorded in the soil samples affected by decaying wood.

## Discussion

The conducted research confirmed the differences between decaying wood and the soil environment in terms of C:N:P stoichiometry. Decaying wood is very rich in C and at the same time poor in terms of N and P. The consequence of this is wide C/N and C/P ratios in decaying wood. The obtained results confirm previous research^31^. In the soil environment, the situation is reversed. The surface horizons of humus accumulation reach high carbon contents with relatively high nitrogen and phosphorus content. As a result of the above, the average C/N ratios are 10–20 times lower in soil compared to wood, and the C/P ratios are even 50–100 times lower than those found in wood. The differences found in the stoichiometry of wood in relation to the soil environment coincide with the earlier research^12,31^. The very wide C:N:P ratio in decaying wood is due to the chemical composition of the wood substance itself. Raw wood composed mainly of cellulose and lignin contains C in the amount of approx. 50%, while N and P in raw wood are only about 1.0–1.5% and 0.15–0.20% respectively^32^. The C:N:P proportions depend to some extent on the wood species^31^. Our experiment was limited to the use of decomposing spruce wood and its impact on the soil environment was analysed. With the advancement of the wood decomposition process, its chemical composition changes^27,33,34^. As a result of the decomposition of polysaccharides, the amount of carbon decreases, while the amount of nitrogen and phosphorus increases relatively^35^. An increase in the content of nitrogen and phosphorus in decomposed wood may be related to the transport of these nutrients by mycelial hyphae from the soil environment adjacent to deadwood^36,37^. Specialized groups of bacteria with the ability to bind atmospheric N also participate in the process of wood decomposition^38,39^.

The experiment confirmed the significant influence of decaying wood on the stoichiometry of soil affected by deadwood. Decaying wood releases large amounts of dissolved carbon, which moves to the underlying soil with fallout^40–42^. Along with leachates from decaying wood, large amounts of nitrates and phosphates are released, which feed the soil under the wood^5^. In our experiment, the most decomposed wood (in V DC) had the strongest impact on the soil, which is consistent with the results of previous studies^41^. The significantly higher nitrogen content in the soil, compared to the control variant (without the influence of wood) was found in all localization conditions under decaying wood in V DC. In the case of phosphorus, we did not find such an unambiguous effect. More phosphorus than controls accumulated under the V DC wood on most of the tested surfaces but there were exceptions to this rule. At the cooler exhibition, such exceptions occurred at an altitude of 800 and 1000 m above sea level, at the highest altitude at the warmer exposure. Phosphorus is considered to be an ingredient limiting the development of microorganisms decomposing organic matter^43,44^. There are reports of a higher accumulation of this macroelement in cooler locations, where the growing season is shorter and probably the entire P pool is not used by soil microorganisms^45,46^.

In our research, the GLM analysis confirmed the importance of the influence of altitude and slope exposure on the formation of the C:N:P stoichiometry. In mountainous areas, the climatic conditions change with the altitude, the temperature decreases and the amount of precipitation increases at the same time. According to Allison et al.^47,48^, temperature could influence decomposition of organic material by changing the activity of the decomposer community and also by changing plant species composition and litter chemistry. Climatic factors slow down biological activity and they explain the morphology of the humus forms and their variations^49^. In the area of our experiments, in the highest locations (1200 m above sea level), the vegetation period is shorter by 50 days, and rainfall is on average 200 mm higher than in the lowest locations per year^30,50^. In the highest locations, a significant slowdown in the rate of decomposition of deadwood can be expected and, at the same time, increased processes of washing the decomposition products into the soil beneath the wood. In our study, we found an expansion of the C:P ratio in wood in III and IV DC with increasing altitude, culminating at 1000 m above sea level. In the case of the most decomposed wood, the C:P ratio decreased with increasing altitude. On the other hand, in soil influenced by wood, the C:P ratio tends to increase, as in less decomposed wood. In wood, in the earlier stages of decomposition, we observed a decrease in the content of phosphorus with increasing altitude, which proves its role as a component occurring in the minimum, determining the occurrence of biochemical processes in the soil, including the processes of decomposition of organic matter^43,44^. It does not indicate the presence of an excess of this macroelement in high positions as suggested^51^. At the same time, in the most decomposed wood, the content of phosphorus increases, which can be explained by the differences in the time needed to reach a certain stage of decomposition in different climatic conditions. Herrmann and Bauhus^35^ proved that in the case of spruce logs, after the loss of approx. 40% of mass, the P content increases rapidly and is the greater the longer the decomposition process. For the N:P ratio, we found very similar ranges in the wood and in the affected soil. In most cases, they are in the range of 40–80. In deadwood, they show an upward trend with height up to 1000 m above sea level, a downward trend is observed in higher areas. In the soil, N:P first narrows, then after exceeding the position of 800–1000 m above sea level, it shows an upward trend again. It is difficult to explain the observed trends clearly. In studies carried out in various regions of Germany, significantly lower N/P ratios were found in spruce CWD^35^. In the initial stages of decomposition, this ratio was 13–23, while spruce wood, more strongly decomposed, increased the N/P ratio to the value of approx. 30, with wood decomposing mainly in lowlands and upland areas.

## Conclusions

Our research confirmed the importance of the location conditions and the degree of decomposition in shaping the C/N/P stoichiometry in the deadwood-soil system. Deadwood, regardless of the degree of decomposition, was characterized by a higher C/N/P stoichiometry compared to soils. The C, N and P stoichiometry of mountain soils was most strongly affected by wood in the 5th degree of decomposition, regardless of location. With the height of a.s.l. the C/N and C/P ratio increases, which is associated with a change in climate conditions. The obtained results indicate a positive effect of decaying wood on the C/N/P stoichiometry. The research provides justification for leaving wood to natural decomposition in forest ecosystems in order to improve biogeochemical cycles. The positive impact of decaying wood on soil properties will result in improved stability of forest ecosystems.

## Data Availability

Data will be made available on request. In order to receive data from this study, please contact Ewa Błońska: ewa.blonska@urk.edu.pl.
